# Supplementary material for: Associations between Conventional and Emerging Indicators of Dietary Carbohydrate Quality and New-Onset Type 2 Diabetes Mellitus in Chinese Adults
Source: Nutrients. 2023 Jan 27;15(3):647. doi: 10.3390/nu15030647 (PMC9919288; doi:10.3390/nu15030647)
Supplement: Supplementary file 1 [file nutrients-15-00647-s001.zip › Table S1 and Table S2-proofed.pdf]

Table S1. Associations between dietary GI, CF and CQI values and T2DM risk in 14590 Chinese adults of the China Health and Nutrition Survey 1997 - 2015, stratified by region, smoking status, alcohol consumption, physical activity status, dietary intake of total energy, total carbohydrate and cholesterol [1].

Variables	N	Cases (incidence rate, ‰ person-year)	HR (95% CI) of quintiles of carbohydrate quality indicators					*P-trend*	*P-interaction*
			Q1	Q2	Q3	Q4	Q5		
**Dietary GI**									
Region									
Northern	6066	538 (9.32)	1.00 (Ref)	0.70 (0.44, 1.11)	0.49 (0.30, 0.78)	0.43 (0.25, 0.75)	0.71 (0.35, 1.43)	0.0081	0.27
Southern	8524	515 (5.99)	1.00 (Ref)	1.12 (0.51, 2.47)	0.81 (0.38, 1.72)	0.76 (0.36, 1.61)	0.74 (0.35, 1.59)	0.21	
Smoking status									
No	9133	654 (7.84)	1.00 (Ref)	0.65 (0.43, 0.98)	0.47 (0.31, 0.72)	0.45 (0.29, 0.70)	0.56 (0.35, 0.91)	0.0074	0.71
Yes	5457	399 (6.63)	1.00 (Ref)	0.94 (0.50, 1.75)	0.82 (0.44, 1.52)	0.53 (0.28, 1.01)	0.72 (0.36, 1.44)	0.0006	
Alcohol consumption									
No	7478	546 (8.40)	1.00 (Ref)	0.58 (0.37, 0.92)	0.43 (0.27, 0.68)	0.40 (0.25, 0.66)	0.54 (0.32, 0.90)	0.0094	0.27
Yes	7112	507 (6.45)	1.00 (Ref)	0.98 (0.58, 1.64)	0.78 (0.46, 1.32)	0.62 (0.36, 1.07)	0.75 (0.41, 1.36)	0.10	
Physical activity status, median, METs-h/week									
< 90.79	5945	481 (8.54)	1.00 (Ref)	0.70 (0.44, 1.14)	0.55 (0.33, 0.92)	0.57 (0.33, 1.00)	0.69 (0.37, 1.29)	0.15	0.88
≥ 90.79	8645	572 (6.55)	1.00 (Ref)	0.80 (0.53, 1.23)	0.63 (0.41, 0.95)	0.46 (0.30, 0.71)	0.62 (0.39, 0.97)	0.0071	
Dietary total energy intake, median, kcal/d									
< 2105.44	7295	471 (7.01)	1.00 (Ref)	0.48 (0.26, 0.90)	0.44 (0.23, 0.85)	0.30 (0.16, 0.59)	0.56 (0.29, 1.08)	0.1407	0.18
≥ 2105.44	7295	582 (7.61)	1.00 (Ref)	0.55 (0.33, 0.93)	0.49 (0.30, 0.81)	0.36 (0.20, 0.65)	0.41 (0.22, 0.79)	0.0032	
Dietary total carbohydrate intake, median, % energy									
< 55.46	7295	480 (7.39)	1.00 (Ref)	0.60 (0.42, 0.88)	0.56 (0.38, 0.83)	0.61 (0.39, 0.94)	1.06 (0.64, 1.75)	0.56	0.15
≥ 55.46	7295	573 (7.29)	1.00 (Ref)	0.82 (0.53, 1.27)	0.64 (0.41, 0.98)	0.52 (0.34, 0.79)	0.65 (0.42, 1.00)	0.0141	
Dietary cholesterol intake, median, mg/d									
< 127.85	7295	534 (7.17)	1.00 (Ref)	0.65 (0.45, 0.93)	0.58 (0.41, 0.82)	0.52 (0.37, 0.74)	0.66 (0.47, 0.93)	0.06	0.45
≥ 127.85	7295	519 (7.51)	1.00 (Ref)	0.63 (0.49, 0.82)	0.51 (0.39, 0.68)	0.48 (0.35, 0.67)	0.87 (0.61, 1.24)	0.0157	

Table S1 (Continued)

**Variables**	**N**	**Cases (incidence rate, ‰ person-year)**	**HR (95% CI) of quintiles of carbohydrate quality indicators**					***P*-trend**	***P*-interaction**
			**Q1**	**Q2**	**Q3**	**Q4**	**Q5**		
**CF**									
Region									
Northern	6066	538 (9.32)	1.00 (Ref)	0.63 (0.43, 0.93)	0.54 (0.34, 0.84)	0.44 (0.25, 0.79)	0.61 (0.30, 1.26)	0.11	0.93
Southern	8524	515 (5.99)	1.00 (Ref)	0.87 (0.62, 1.20)	1.10 (0.71, 1.70)	1.15 (0.64, 2.07)	1.78 (0.87, 3.63)	0.15	
Smoking status									
No	9133	654 (7.84)	1.00 (Ref)	0.66 (0.50, 0.87)	0.68 (0.49, 0.95)	0.63 (0.41, 0.97)	1.01 (0.60, 1.70)	0.74	0.83
Yes	5457	399 (6.63)	1.00 (Ref)	0.80 (0.52, 1.23)	0.76 (0.44, 1.30)	0.66 (0.32, 1.36)	1.03 (0.41, 2.57)	0.84	
Alcohol consumption									
No	7478	546 (8.40)	1.00 (Ref)	0.65 (0.47, 0.91)	0.79 (0.54, 1.15)	0.70 (0.43, 1.15)	1.02 (0.56, 1.85)	0.91	0.71
Yes	7112	507 (6.45)	1.00 (Ref)	0.75 (0.53, 1.07)	0.66 (0.42, 1.04)	0.60 (0.32, 1.10)	0.95 (0.44, 2.07)	0.65	
Physical activity status, median, METs-h/week									
< 90.79	5945	481 (8.54)	1.00 (Ref)	0.70 (0.49, 1.00)	0.77 (0.49, 1.22)	0.65 (0.36, 1.20)	0.79 (0.37, 1.70)	0.55	0.86
≥ 90.79	8645	572 (6.55)	1.00 (Ref)	0.72 (0.53, 0.98)	0.71 (0.50, 1.03)	0.68 (0.42, 1.10)	1.12 (0.62, 2.00)	0.98	
Dietary total energy intake, median, kcal/d									
< 2105.44	7295	471 (7.01)	1.00 (Ref)	0.65 (0.45, 0.92)	0.68 (0.45, 1.04)	0.60 (0.35, 1.05)	1.18 (0.61, 2.29)	0.95	0.25
≥ 2105.44	7295	582 (7.61)	1.00 (Ref)	0.72 (0.51, 1.03)	0.76 (0.50, 1.15)	0.60 (0.34, 1.06)	0.85 (0.42, 1.72)	0.55	
Dietary total carbohydrate intake, median, % energy									
< 55.46	7295	480 (7.39)	1.00 (Ref)	0.66 (0.48, 0.92)	0.77 (0.50, 1.18)	0.71 (0.40, 1.26)	0.87 (0.42, 1.81)	0.51	0.94
≥ 55.46	7295	573 (7.29)	1.00 (Ref)	0.78 (0.53, 1.14)	0.64 (0.41, 1.00)	0.57 (0.32, 1.01)	0.83 (0.41, 1.65)	0.58	
Dietary cholesterol intake, median, mg/d									
< 127.85	7295	534 (7.17)	1.00 (Ref)	0.81 (0.58, 1.12)	0.84 (0.56, 1.25)	0.83 (0.49, 1.41)	1.19 (0.63, 2.27)	0.68	0.86
≥ 127.85	7295	519 (7.51)	1.00 (Ref)	0.65 (0.47, 0.90)	0.68 (0.46, 1.01)	0.60 (0.36, 1.01)	1.01 (0.53, 1.93)	0.66	

Table S1 (Continued)

Variables	N	Cases (incidence rate, ‰ person-years)	HR (95% CI) of quintiles of carbohydrate quality indicators					*P-trend*	*P-interaction*
			Q1	Q2	Q3	Q4	Q5		
**CQI**									
Region									
Northern	6066	538 (9.32)	1.00 (Ref)	1.03 (0.74, 1.43)	0.94 (0.68, 1.30)	0.89 (0.66, 1.21)	0.76 (0.54, 1.06)	0.07	0.29
Southern	8524	515 (5.99)	1.00 (Ref)	0.68 (0.46, 0.98)	0.71 (0.51, 0.99)	0.83 (0.61, 1.12)	0.73 (0.54, 0.98)	0.21	
Smoking status									
No	9133	654 (7.84)	1.00 (Ref)	0.96 (0.71, 1.30)	0.95 (0.71, 1.26)	0.93 (0.72, 1.21)	0.89 (0.68, 1.15)	0.36	0.17
Yes	5457	399 (6.63)	1.00 (Ref)	0.74 (0.51, 1.09)	0.61 (0.43, 0.88)	0.71 (0.51, 0.99)	0.50 (0.35, 0.71)	0.0006	
Alcohol consumption									
No	7478	546 (8.40)	1.00 (Ref)	0.93 (0.67, 1.29)	0.81 (0.59, 1.10)	0.98 (0.74, 1.30)	0.80 (0.59, 1.07)	0.24	0.74
Yes	7112	507 (6.45)	1.00 (Ref)	0.81 (0.57, 1.15)	0.80 (0.58, 1.11)	0.71 (0.52, 0.96)	0.65 (0.48, 0.89)	0.0057	
Physical activity status, median, METs-h/week									
< 90.79	5945	481 (8.54)	1.00 (Ref)	1.07 (0.76, 1.51)	0.96 (0.69, 1.33)	0.80 (0.58, 1.10)	0.80 (0.59, 1.09)	0.0387	0.27
≥ 90.79	8645	572 (6.55)	1.00 (Ref)	0.74 (0.54, 1.03)	0.74 (0.55, 1.00)	0.87 (0.67, 1.13)	0.66 (0.50, 0.87)	0.0357	
Dietary total energy intake, median, kcal/d									
< 2105.44	7295	471 (7.01)	1.00 (Ref)	1.04 (0.75, 1.45)	0.88 (0.64, 1.22)	0.84 (0.61, 1.14)	0.73 (0.53, 1.01)	0.0389	0.63
≥ 2105.44	7295	582 (7.61)	1.00 (Ref)	0.69 (0.49, 0.98)	0.71 (0.52, 0.98)	0.77 (0.58, 1.03)	0.62 (0.46, 0.84)	0.0141	
Dietary total carbohydrate intake, median, % energy									
< 55.46	7295	480 (7.39)	1.00 (Ref)	0.77 (0.54, 1.09)	0.77 (0.56, 1.07)	0.78 (0.58, 1.06)	0.68 (0.50, 0.93)	0.0402	0.85
≥ 55.46	7295	573 (7.29)	1.00 (Ref)	1.03 (0.76, 1.41)	0.90 (0.67, 1.23)	0.92 (0.70, 1.21)	0.78 (0.59, 1.04)	0.13	
Dietary cholesterol intake, median, mg/d									
< 127.85	7295	534 (7.17)	1.00 (Ref)	1.15 (0.84, 1.60)	0.84 (0.61, 1.17)	0.99 (0.75, 1.32)	0.83 (0.62, 1.11)	0.13	< 0.0001
≥ 127.85	7295	519 (7.51)	1.00 (Ref)	0.66 (0.47, 0.92)	0.87 (0.65, 1.18)	0.78 (0.59, 1.04)	0.72 (0.53, 0.97)	0.13	

[1] Data were presented as HR (95% CI) estimated by using Cox proportional hazard regression models. Adjusted confounders included age, sex, education level, urbanization index,

region, smoking status, alcohol consumption, BMI, physical activity status, total energy, cholesterol and PUFA to SFA ratio in models of CF and CQI. Total carbohydrate and fiber intakes were additionally adjusted in models of dietary GI. Abbreviations: BMI, body mass index; CF, carbohydrate to fiber ratio; CI, confidence intervals; CQI: carbohydrate quality index; GI, glycemic index; HR, hazard ratio; MET: metabolic equivalent task hour; PUFA, polyunsaturated fatty acid; Q, quintiles; SFA, saturated fatty acid; T2DM, type 2 diabetes mellitus.

Table S2. Associations between dietary GI, CF and T2DM risk in 14590 Chinese adults of the China Health and Nutrition Survey 1997 – 2015, stratified by age, sex, BMI, baseline hypertension, urbanization index, education level, PUFA: SFA, region, smoking status, alcohol consumption, physical activity status, dietary intake of energy, total carbohydrate and cholesterol [1].

Variables	N	Cases (incidence rate, ‰ person-year)	HR (95% CI) of quintiles of carbohydrate quality indicators					*P-trend*	*P-interaction*
			Q1	Q2	Q3	Q4	Q5		
**Dietary GI**									
Age									
< 60 y	11881	787 (6.38)	1.80 (1.40, 2.32)	1.25 (0.99, 1.58)	1.11 (0.89, 1.38)	1.00 (Ref)	1.34 (1.07, 1.68)	0.0162	0.47
≥ 60 y	2709	266 (13.13)	1.87 (1.23, 2.85)	1.25 (0.84, 1.87)	0.79 (0.51, 1.22)	1.00 (Ref)	1.30 (0.85, 1.98)	0.0310	
Sex									
Male	7402	517 (6.92)	1.86 (1.34, 2.59)	1.15 (0.85, 1.55)	1.12 (0.85, 1.49)	1.00 (Ref)	1.44 (1.07, 1.92)	0.18	0.56
Female	7188	536 (7.77)	1.99 (1.45, 2.74)	1.35 (1.01, 1.79)	0.96 (0.72, 1.29)	1.00 (Ref)	1.20 (0.89, 1.61)	0.0007	
BMI, kg/m^2									
< 24.0	9313	423 (4.51)	2.01 (1.39, 2.92)	1.55 (1.10, 2.17)	1.33 (0.96, 1.86)	1.00 (Ref)	1.89 (1.36, 2.65)	0.22	0.0259
≥ 24.0	5277	630 (12.62)	2.08 (1.55, 2.77)	1.16 (0.89, 1.52)	0.98 (0.76, 1.26)	1.00 (Ref)	1.08 (0.82, 1.41)	0.0002	
Baseline hypertension									
No	11495	683 (5.80)	1.88 (1.40, 2.51)	1.34 (1.04, 1.73)	1.23 (0.97, 1.58)	1.00 (Ref)	1.35 (1.04, 1.74)	0.0116	0.52
Yes	3095	370 (14.26)	1.78 (1.20, 2.66)	1.23 (0.84, 1.79)	0.88 (0.60, 1.28)	1.00 (Ref)	1.23 (0.85, 1.77)	0.0453	
Urbanization index, median									
< 69.46	7293	491 (5.86)	2.34 (1.43, 3.83)	1.78 (1.19, 2.66)	1.31 (0.90, 1.90)	1.00 (Ref)	1.06 (0.74, 1.50)	0.0004	0.0068
≥ 69.46	7297	562 (9.40)	1.96 (1.23, 3.13)	1.14 (0.75, 1.75)	1.10 (0.73, 1.64)	1.00 (Ref)	2.27 (1.38, 3.76)	0.75	
Education level									
Primary or lower	6860	618 (8.22)	2.17 (1.36, 3.45)	1.85 (1.22, 2.81)	1.16 (0.79, 1.70)	1.00 (Ref)	1.16 (0.79, 1.71)	0.0016	0.29
Middle or above	7730	435 (6.35)	1.89 (1.14, 3.15)	1.24 (0.79, 1.96)	1.33 (0.87, 2.02)	1.00 (Ref)	1.92 (1.21, 3.05)	0.72	
Dietary PUFA: SFA, median									
< 1.15	7295	479 (6.75)	1.55 (1.12, 2.15)	1.02 (0.75, 1.39)	0.97 (0.71, 1.30)	1.00 (Ref)	1.64 (1.17, 2.30)	0.74	0.0069
≥ 1.15	7295	574 (7.90)	2.19 (1.58, 3.03)	1.59 (1.19, 2.12)	1.27 (0.96, 1.68)	1.00 (Ref)	1.21 (0.92, 1.58)	< 0.0001	

Table S2 (Continued)

Variables	N	Cases (incidence rate, ‰ person-year)	HR (95% CI) of quintiles of carbohydrate quality indicators					*P-trend*	*P-interaction*
			Q1	Q2	Q3	Q4	Q5		
Region									
Northern	6066	538 (9.32)	2.32 (1.33, 4.03)	1.62 (0.95, 2.79)	1.13 (0.67, 1.92)	1.00 (Ref)	1.65 (0.81, 3.37)	0.0081	0.27
Southern	8524	515 (5.99)	1.32 (0.62, 2.79)	1.48 (0.83, 2.62)	1.06 (0.67, 1.67)	1.00 (Ref)	0.98 (0.65, 1.46)	0.21	
Smoking status									
No	9133	654 (7.84)	2.23 (1.43, 3.46)	1.45 (0.96, 2.19)	1.06 (0.72, 1.54)	1.00 (Ref)	1.26 (0.84, 1.87)	0.0074	0.71
Yes	5457	399 (6.63)	1.88 (0.99, 3.60)	1.76 (1.03, 3.03)	1.54 (0.94, 2.52)	1.00 (Ref)	1.35 (0.82, 2.23)	0.11	
Alcohol consumption									
No	7478	546 (8.40)	2.48 (1.52, 4.06)	1.44 (0.89, 2.31)	1.06 (0.68, 1.64)	1.00 (Ref)	1.33 (0.85, 2.07)	0.0094	0.27
Yes	7112	507 (6.45)	1.62 (0.93, 2.80)	1.58 (1.01, 2.47)	1.26 (0.84, 1.91)	1.00 (Ref)	1.20 (0.78, 1.86)	0.10	
Physical activity status, median, METs-h/week									
< 90.79	5945	481 (8.54)	1.74 (1.00, 3.06)	1.23 (0.76, 1.99)	0.96 (0.60, 1.54)	1.00 (Ref)	1.21 (0.70, 2.08)	0.15	0.88
≥ 90.79	8645	572 (6.55)	2.18 (1.42, 3.36)	1.75 (1.18, 2.59)	1.36 (0.96, 1.94)	1.00 (Ref)	1.35 (0.95, 1.91)	0.0071	
Dietary total energy intake, median, kcal/d									
< 2105.44	7295	471 (7.01)	3.30 (1.69, 6.44)	1.59 (0.90, 2.81)	1.47 (0.85, 2.52)	1.00 (Ref)	1.84 (1.08, 3.12)	0.14	0.18
≥ 2105.44	7295	582 (7.61)	2.75 (1.55, 4.89)	1.53 (0.91, 2.56)	1.35 (0.84, 2.17)	1.00 (Ref)	1.14 (0.67, 1.95)	0.0032	
Dietary total carbohydrate intake, median, % energy									
< 55.46	7295	480 (7.39)	1.65 (1.06, 2.54)	0.99 (0.65, 1.53)	0.92 (0.61, 1.40)	1.00 (Ref)	1.74 (1.07, 2.82)	0.56	0.15
≥ 55.46	7295	573 (7.29)	1.92 (1.26, 2.93)	1.58 (1.11, 2.24)	1.22 (0.88, 1.69)	1.00 (Ref)	1.24 (0.91, 1.69)	0.0141	
Dietary cholesterol intake, median, mg/d									
< 127.85	7295	534 (7.17)	1.91 (1.36, 2.67)	1.24 (0.92, 1.66)	1.10 (0.84, 1.44)	1.00 (Ref)	1.26 (0.98, 1.63)	0.06	0.45
≥ 127.85	7295	519 (7.51)	2.07 (1.49, 2.87)	1.30 (0.96, 1.78)	1.06 (0.78, 1.45)	1.00 (Ref)	1.80 (1.25, 2.59)	0.0157	

Table S2 (Continued)

Variables	N	Cases (incidence rate, ‰ person-year)	HR (95% CI) of quintiles of carbohydrate quality indicators					*P*-trend	*P*-interaction
			Q1	Q2	Q3	Q4	Q5		
**CF**									
Age									
< 60 y	11881	787 (6.38)	1.35 (0.88, 2.06)	1.13 (0.80, 1.58)	1.05 (0.81, 1.35)	1.00 (Ref)	1.77 (1.37, 2.27)	0.64	0.33
≥ 60 y	2709	266 (13.13)	2.43 (1.26, 4.67)	1.15 (0.64, 2.06)	1.13 (0.72, 1.76)	1.00 (Ref)	1.03 (0.67, 1.59)	0.0247	
Sex									
Male	7402	517 (6.92)	1.58 (0.92, 2.74)	1.13 (0.72, 1.76)	0.98 (0.70, 1.37)	1.00 (Ref)	1.34 (0.98, 1.85)	0.48	0.68
Female	7188	536 (7.77)	1.48 (0.90, 2.44)	1.09 (0.71, 1.65)	1.21 (0.88, 1.66)	1.00 (Ref)	1.76 (1.28, 2.43)	0.84	
BMI, kg/m^2									
< 24.0	9313	423 (4.51)	1.73 (0.97, 3.09)	1.37 (0.85, 2.20)	0.90 (0.63, 1.30)	1.00 (Ref)	1.03 (0.73, 1.45)	0.11	0.0081
≥ 24.0	5277	630 (12.62)	1.51 (0.94, 2.42)	1.00 (0.67, 1.48)	1.20 (0.89, 1.61)	1.00 (Ref)	2.13 (1.58, 2.88)	0.52	
Baseline hypertension									
No	11495	683 (5.80)	1.63 (1.01, 2.63)	1.23 (0.83, 1.80)	1.11 (0.83, 1.47)	1.00 (Ref)	1.43 (1.08, 1.90)	0.41	0.48
Yes	3095	370 (14.26)	1.67 (0.90, 3.11)	1.20 (0.71, 2.02)	1.23 (0.81, 1.85)	1.00 (Ref)	1.80 (1.21, 2.68)	0.90	
Urbanization index, median									
< 69.46	7293	491 (5.86)	1.75 (0.92, 3.34)	1.21 (0.74, 1.96)	1.05 (0.74, 1.48)	1.00 (Ref)	1.70 (1.20, 2.40)	0.88	0.44
≥ 69.46	7297	562 (9.40)	1.42 (0.89, 2.27)	0.98 (0.66, 1.47)	1.12 (0.82, 1.54)	1.00 (Ref)	1.22 (0.90, 1.66)	0.51	
Education level									
Primary or lower	6860	618 (8.22)	1.93 (1.16, 3.22)	1.22 (0.81, 1.83)	1.16 (0.86, 1.57)	1.00 (Ref)	1.39 (1.03, 1.86)	0.27	0.32
Middle or above	7730	435 (6.35)	1.22 (0.71, 2.10)	0.99 (0.63, 1.58)	1.08 (0.75, 1.55)	1.00 (Ref)	1.65 (1.15, 2.35)	0.63	
Dietary PUFA: SFA, median									
< 1.15	7295	479 (6.75)	1.44 (0.85, 2.47)	1.21 (0.78, 1.89)	1.15 (0.81, 1.64)	1.00 (Ref)	1.34 (0.97, 1.84)	0.64	0.40
≥ 1.15	7295	574 (7.90)	1.76 (1.04, 2.99)	1.11 (0.73, 1.71)	1.14 (0.84, 1.56)	1.00 (Ref)	1.90 (1.35, 2.68)	0.75	

Table S2 (Continued)

**Variables**	**N**	**Cases (incidence rate, ‰ person-years)**	**HR (95% CI) of quintiles of carbohydrate quality indicators**					***P-trend***	***P-interaction***
			**Q1**	**Q2**	**Q3**	**Q4**	**Q5**		
Region									
Northern	6066	538 (9.32)	2.25 (1.27, 3.99)	1.43 (0.89, 2.30)	1.21 (0.84, 1.74)	1.00 (Ref)	1.38 (1.01, 1.89)	0.11	0.93
Southern	8524	515 (5.99)	0.87 (0.48, 1.57)	0.76 (0.48, 1.20)	0.96 (0.68, 1.35)	1.00 (Ref)	1.56 (1.04, 2.33)	0.15	
Smoking status									
No	9133	654 (7.84)	1.59 (1.04, 2.45)	1.05 (0.73, 1.52)	1.08 (0.82, 1.44)	1.00 (Ref)	1.61 (1.22, 2.13)	0.74	0.83
Yes	5457	399 (6.63)	1.51 (0.74, 3.12)	1.21 (0.70, 2.10)	1.14 (0.77, 1.69)	1.00 (Ref)	1.55 (1.05, 2.30)	0.84	
Alcohol consumption									
No	7478	546 (8.40)	1.42 (0.87, 2.33)	0.93 (0.61, 1.41)	1.12 (0.82, 1.54)	1.00 (Ref)	1.45 (1.05, 1.99)	0.91	0.71
Yes	7112	507 (6.45)	1.68 (0.91, 3.10)	1.27 (0.79, 2.04)	1.10 (0.78, 1.57)	1.00 (Ref)	1.60 (1.14, 2.25)	0.65	
Physical activity status, median, METs-h/week									
< 90.79	5945	481 (8.54)	1.53 (0.84, 2.79)	1.07 (0.67, 1.71)	1.18 (0.84, 1.67)	1.00 (Ref)	1.21 (0.86, 1.71)	0.55	0.86
≥ 90.79	8645	572 (6.55)	1.47 (0.91, 2.39)	1.06 (0.70, 1.59)	1.05 (0.77, 1.43)	1.00 (Ref)	1.64 (1.21, 2.23)	0.98	
Dietary total energy intake, median, kcal/d									
< 2105.44	7295	471 (7.01)	1.66 (0.96, 2.89)	1.07 (0.67, 1.71)	1.13 (0.78, 1.64)	1.00 (Ref)	1.96 (1.37, 2.81)	0.95	0.25
≥ 2105.44	7295	582 (7.61)	1.65 (0.94, 2.92)	1.20 (0.76, 1.88)	1.25 (0.90, 1.73)	1.00 (Ref)	1.40 (1.02, 1.94)	0.55	
Dietary total carbohydrate intake, median, % energy									
< 55.46	7295	480 (7.39)	1.41 (0.79, 2.52)	0.94 (0.58, 1.51)	1.09 (0.75, 1.57)	1.00 (Ref)	1.23 (0.83, 1.82)	0.51	0.94
≥ 55.46	7295	573 (7.29)	1.76 (0.99, 3.11)	1.37 (0.88, 2.12)	1.12 (0.81, 1.54)	1.00 (Ref)	1.45 (1.07, 1.98)	0.58	
Dietary cholesterol intake, median, mg/d									
< 127.85	7295	534 (7.17)	1.20 (0.71, 2.03)	0.97 (0.64, 1.47)	1.01 (0.74, 1.37)	1.00 (Ref)	1.44 (1.04, 1.99)	0.68	0.86
≥ 127.85	7295	519 (7.51)	1.67 (0.99, 2.81)	1.09 (0.70, 1.69)	1.13 (0.81, 1.59)	1.00 (Ref)	1.68 (1.23, 2.31)	0.66	

[1] Data were presented as HR (95% CI) estimated by using Cox proportional hazard regression models. Reference group was the Q4 of dietary GI or CF. Adjusted confounders included age, sex, education level, urbanization index, region, smoking status, alcohol consumption, BMI, physical activity status, total energy, cholesterol and PUFA to SFA ratio in models of CF. Total

carbohydrate and fiber intakes were additionally adjusted in models of dietary GI. Abbreviations: BMI, body mass index; CF, carbohydrate to fiber ratio; CI, confidence intervals; GI, glycemic index; HR, hazard ratio; MET: metabolic equivalent task hour; PUFA, polyunsaturated fatty acid; Q, quintiles; SFA, saturated fatty acid; T2DM, type 2 diabetes mellitus.
