# Supplementary figures and images for: Associations between Conventional and Emerging Indicators of Dietary Carbohydrate Quality and New-Onset Type 2 Diabetes Mellitus in Chinese Adults
Source: Nutrients. 2023 Jan 27;15(3):647. doi: 10.3390/nu15030647 (PMC9919288; doi:10.3390/nu15030647)

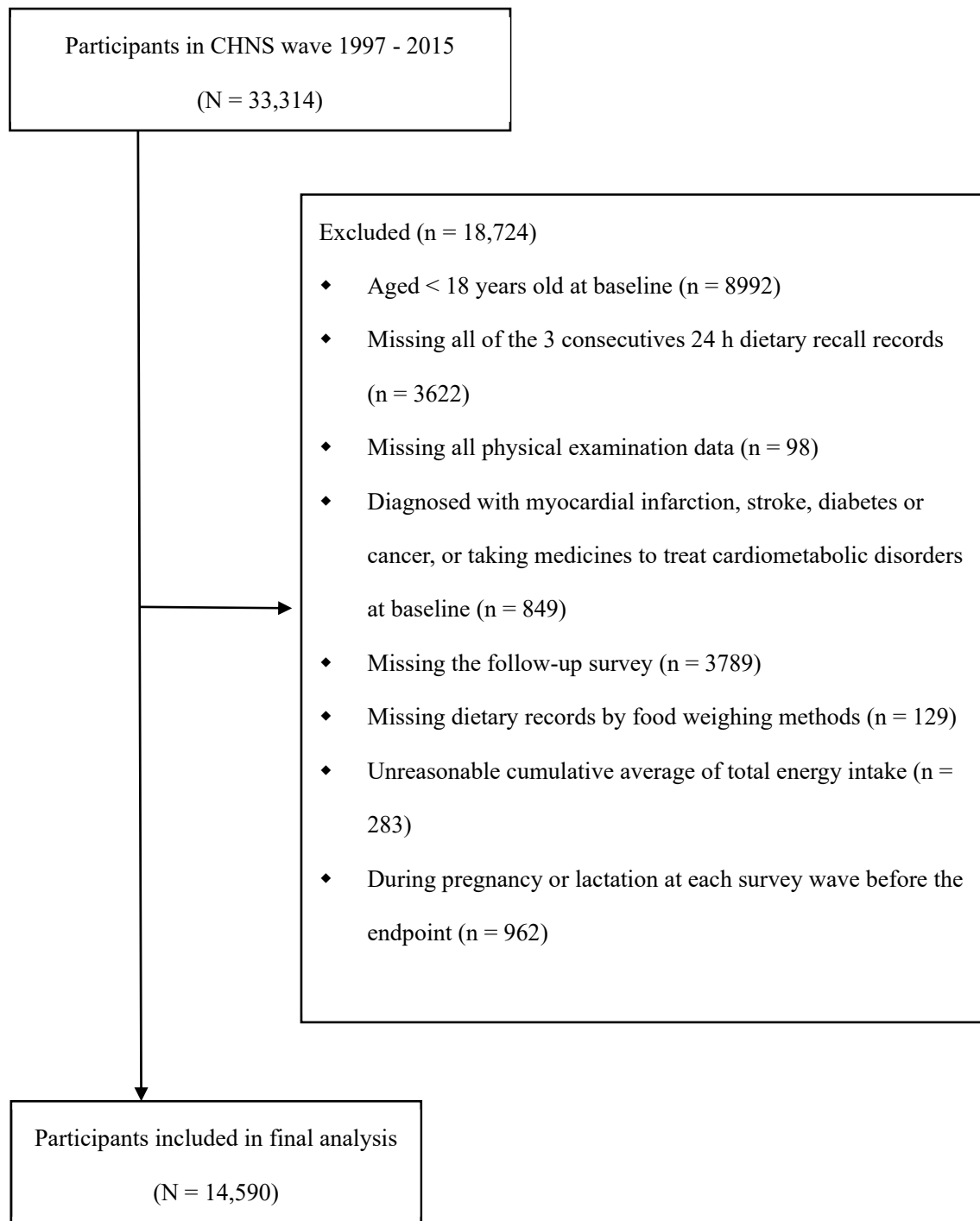

**Figure S1. Flow chart of the 14,590 Chinese adults of the China Health and Nutrition Survey (CHNS) 1997 – 2015.**

Supplement: Supplementary file 1 [file nutrients-15-00647-s001.zip › Figure S1-proofed.pdf]
